# Antiherpetic drugs: a potential way to prevent Alzheimer’s disease?

**DOI:** 10.1186/s13195-021-00950-0

**Published:** 2022-01-07

**Authors:** Morgane Linard, Julien Bezin, Emilie Hucteau, Pierre Joly, Isabelle Garrigue, Jean-François Dartigues, Antoine Pariente, Catherine Helmer

**Affiliations:** 1 University of Bordeaux, Inserm, Bordeaux Population Health Research Center, UMR U1219, F-33000 Bordeaux, France; 2 Pharmacology Department, Bordeaux University Hospital, F-33076 Bordeaux, France; 3 Virology Department, Bordeaux University Hospital and University of Bordeaux, CNRS-UMR 5234, F-33000 Bordeaux, France; 4 Memory Consultation, CMRR, Bordeaux University Hospital, F-33076 Bordeaux, France

**Keywords:** Herpesvirus, Alzheimer’s disease, Vascular dementia, Dementia, Prevention, Antiherpetic drugs, Medico-administrative databases, Infection, Antimicrobial, Treatment

## Abstract

**Background:**

Considering the growing body of evidence suggesting a potential implication of herpesviruses in the development of dementia, several authors have questioned a protective effect of antiherpetic drugs (AHDs) which may represent a new means of prevention, well tolerated and easily accessible. Subsequently, several epidemiological studies have shown a reduction in the risk of dementia in subjects treated with AHDs, but the biological plausibility of this association and the impact of potential methodological biases need to be discussed in more depth.

**Methods:**

Using a French medico-administrative database, we assessed the association between the intake of systemic AHDs and the incidence of (i) dementia, (ii) Alzheimer’s disease (AD), and (iii) vascular dementia in 68,291 subjects over 65 who were followed between 2009 and 2017. Regarding potential methodological biases, Cox models were adjusted for numerous potential confounding factors (including proxies of sociodemographic status, comorbidities, and use of healthcare) and sensitivity analyses were performed in an attempt to limit the risk of indication and reverse causality biases.

**Results:**

9.7% of subjects (*n*=6642) had at least one intake of systemic AHD, and 8883 incident cases of dementia were identified. Intake of at least one systemic AHD during follow-up was significantly associated with a decreased risk of AD (aHR 0.85 95% confidence interval [0.75–0.96], *p*=0.009) and, to a lesser extent with respect to *p* values, to both dementia from any cause and vascular dementia. The association with AD remained significant in sensitivity analyses. The number of subjects with a *regular* intake was low and prevented us from studying its association with dementia.

**Conclusions:**

Taking at least one systemic AHD during follow-up was significantly associated with a 15% reduced risk of developing AD, even after taking into account several potential methodological biases. Nevertheless, the low frequency of subjects with a regular intake questions the biological plausibility of this association and highlights the limits of epidemiological data to evaluate a potential protective effect of a regular treatment by systemic AHDs on the incidence of dementia

**Supplementary Information:**

The online version contains supplementary material available at 10.1186/s13195-021-00950-0.

## Background

Given the limited therapeutic arsenal available for a disorder as devastating as Alzheimer’s disease (AD), identification of new avenues of prevention is a public health priority. While the majority of therapeutic trials currently focus on the evaluation of anti-amyloid or anti-tau biotherapies, another means of prevention deserves to be explored: the antiherpetic drugs (AHDs).

Indeed, the recent discovery of the antimicrobial role of the amyloid peptide [1–4] reinforced the hypothesis implicating infectious agents in the development of AD. Among the suspected pathogens (which include in particular different herpesviruses [5–7]), herpes simplex virus type 1 (HSV-1) is the most studied candidate [8], and numerous studies in vitro, in animals and in humans provide arguments in favor of its involvement in AD. It is a neurotropic virus with a particular tropism for the temporal lobe. Able to move from neurons to neurons, it is found in the brains of elderly subjects, particularly in areas affected by AD (reviewed in [9]). After infection, usually at a young age [10], it stays in the body in a latent state in the trigeminal ganglion and periodically reactivates, symptomatically or not [11]. Its reactivation leads to the accumulation of pathological hallmarks of AD (including amyloid and tau pathologies as well as neuroinflammation, oxidative stress, mitochondrial damage, impaired autophagy, synaptic dysfunction, and neuronal apoptosis) in vitro or in animal models (reviewed in [12]) and can also translate into the onset of memory-type cognitive decline in mice [13]. With aging, the progressive immunosenescence of both adaptive immunity and microglia may explain (i) an increase in the frequency of viral reactivations in the central nervous system leading to a subsequent accumulation of antimicrobial amyloid peptides and (ii) a decrease in the clearance of these peptides by the microglial cells as well as the appearance of deleterious neuroinflammation for the neurons [14, 15]. Moreover, links can be made between HSV infection and risk factors for AD—especially genetic ones. For example, numerous products of these genetic risk factors seem to interact with HSV during its cellular cycle [16–18] and some of them (including APOE [19–23]) influence the susceptibility of infections and associated complications. Thus, the existence of susceptibility factors (whether genetic, environmental, or related to the virus) may explain why—despite an HSV-1 seroprevalence of around 80% in the elderly [10], some infected subjects remain healthy carriers while others develop neurological complications of the infection.

Consequently, several authors have questioned a protective effect of AHDs, which could represent a new means of prevention, well tolerated and easily accessible. In vitro studies [24–27] have demonstrated that adding AHDs into a cellular medium inhibits the HSV-1-induced appearance of AD markers. Moreover, recent epidemiological results (mainly from medico-administrative databases) suggest a significant reduction in the risk of dementia in subjects infected with HSV or varicella-zoster virus (VZV) and treated with AHDs [28–33]. However, (i) the biological plausibility of the association found and (ii) potential indication or reverse causality biases have rarely been discussed. Thus, with this in mind, we further explored whether the protective effect of systemic AHDs on the onset of dementia was replicable using a French large medico-administrative database.

## Methods

### Data source

The “Echantillon Généraliste des Bénéficiaires” (EGB) is a 1/97th random sample of affiliates to the French Health Insurance System (which covers approximately 98% of the population) [34, 35]. It is representative of the national population in terms of sex and age and contains in particular information relating to (i) sociodemographic data including information of health insurance complementary coverage for low-income people; (ii) “long-term diseases” (LTDs), a group of chronic diseases for which all medical expenses are fully reimbursed; (iii) outpatient healthcare expenditures reimbursements; (iv) outpatient drug reimbursements identified by their Anatomical Therapeutic Chemical (ATC) code and the prescriber specialty [36]; (v) information on hospitalizations with the primary, related, and associate diagnoses coded according to the International Classification of Diseases 10th revision (ICD10); and (vi) dates of death.

### Study design and included subjects

**Fig. 1:**
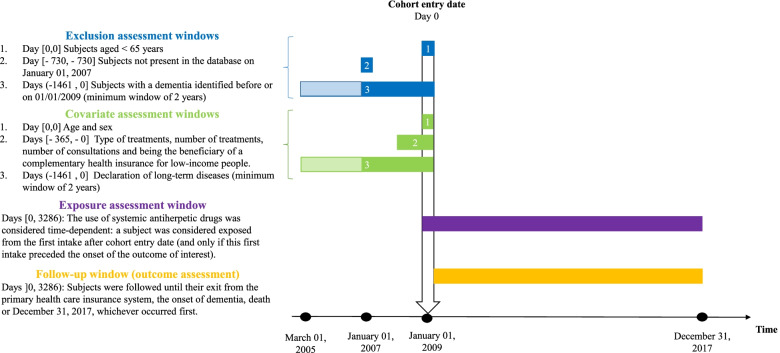
Study design diagram for the main analysis. Figure adapted from the graphic and terminological recommendations of the article by Schneeweiss 2019 [37]

We constituted a cohort study of elderly subjects from the EGB datasource (study design diagram in Fig. 1) [37]. All eligible subjects were included on January 1, 2009 (i.e., cohort entry date) (as the dates of hospitalizations are only available from that date in the EGB). No inclusion took place thereafter.

On January 1, 2009, 507,251 subjects were present in the EGB, and to note, they were all affiliated with the primary health care insurance system (covering approximately 80% of the population in France) as other health insurance schemes joined the EGB later. We then excluded subjects aged under 65 on January 1, 2009 (*n*=433,462), subjects not present in the EGB from January 1, 2007 (*n*=2134) to have a sufficient time window before the cohort entry date to assess exclusion criteria and covariates and subjects with dementia identified before or on January 1, 2009 (*n*=3364 prevalent dementias). The criteria for identifying prevalent dementias are detailed in paragraph 2.4.

**Fig. 2:**
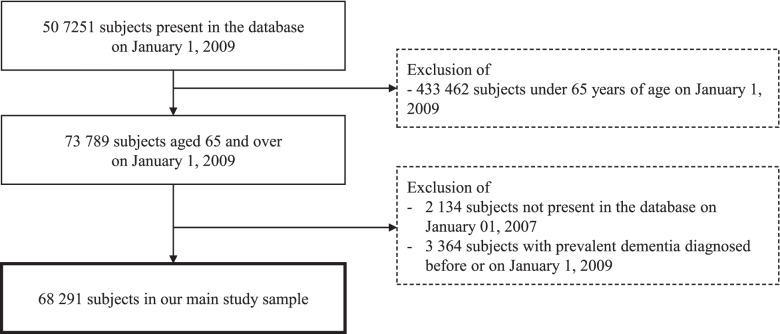
Flow chart of included subjects

Finally, our sample included 68,291 subjects (flow chart in Fig. 2) who were followed until their exit from the primary health care insurance system, the onset of dementia, death, or the end of the study period (December 31, 2017), whichever occurred first.

### Exposure to antiherpetic drugs

During follow-up, we identified all reimbursements for antivirals that were effective against HSV-1 (whether they were usually prescribed for this indication, for cytomegalovirus (CMV) or VZV infections). The ATC codes used to define the intake of AHDs are described in the Additional file 1 and in Table 2 for those used by the subjects in our sample. To assess the association between taking AHDs and the incidence of dementia, the exposure variable was the intake of *systemic* AHDs. Nonsystemic AHDs were not considered since their systemic passage is very low (making an effect on the prevention of dementia unlikely).

For systemic AHDs, we further described the frequency of intake during follow-up, aiming to identify subjects with chronic intake. We assumed that, for a plausible protective effect of AHDs on dementia, the treatment would need to be taken repeatedly. Indeed, it is known that (i) current AHDs are not curative treatments; they only prevent the virus from replicating for the treatment period, (ii) the French recommended durations of treatment are less than 10 days (except in some cases with an occurrence of ≥ 6 herpetic recurrences per year or in some immunocompromised subjects), and (iii) the frequency of asymptomatic reactivations is high (according to Miller et al. 70% of subjects had HSV-1 shedding at least once per month and some more than 6 times per month) [11, 38].

We also identified hospitalizations with diagnoses linked to HSV, VZV, or CMV infections (see Additional file 1) to identify subjects with potentially more severe infections. We could not use the specialty of the prescriber to infer the indication for treatment because the majority of systemic AHDs was prescribed by general practitioners.

### Identification of cases of dementia

Subjects were defined as having (i) dementia from any cause (including unspecified dementia), (ii) AD, or (iii) vascular dementia (VaD) if at least one of the following criteria was present (see Additional file 1):

i) Hospitalization in medical or surgical wards with diagnoses linked to dementia from any cause, AD or VaD.
ii) A declaration of an LTD related to dementia from any cause, AD or VaD.
iii) Anti-dementia drugs (i.e., anti-cholinesterase drugs, memantine, or their association) which were considered only for the identification of dementia from any cause or AD.

Dementia cases identified before or on January 1, 2009, were considered to be prevalent cases (and therefore excluded from the study sample) while those identified after January 01, 2009, were incident cases. For incident cases, the date of dementia onset was defined as the date of hospitalization, the date of declaration of LTD related to dementia or the date of dispensing anti-dementia drugs, whichever occurred first.

### Statistical analysis

Cox proportional hazard regression models were used to estimate cause-specific adjusted hazard ratios (aHRs) and 95% confidence intervals (95% CIs) for all types of dementia and separately for AD and VaD. The use of at least one systemic AHD during follow-up was considered time-dependent (a subject was considered exposed from the first intake after inclusion). Unfortunately, the low frequency of subjects with a regular intake did not allow us to perform analyses distinguishing between occasional and regular intake of AHDs. No analysis by AHD subtype was performed because we had no reason to believe that one treatment would be more effective than another. In adjusted Cox models, several variables were considered potential confounding factors: age, sex, being the beneficiary of a complementary health insurance for low-income people, the presence of several comorbidities (hypertension, diabetes, hypercholesterolemia, stroke, heart diseases), intake of anti-inflammatory drugs, number of outpatient medical consultations the year before inclusion, and number of different medications the year before inclusion (see Additional file 1). Finally, we verified the proportional hazards assumption by testing the existence of an interaction with time.

Several sensitivity analyses were also performed. First, as an increased risk of death was found in subjects treated with at least one systemic AHD (data not shown), we suspected an indication bias due to the prescription of systemic AHDs in contexts with a high mortality rate (immunocompromised subjects or subjects under anticancer chemotherapy). Indeed, the exclusion of immunocompromised subjects or subjects with cancer (see Additional file 1) made the association with death disappear (data not shown). Thus, to avoid a potential bias if the severity of the condition of some subjects treated with AHDs changes the probability of being identified as demented in the database, we performed a sensitivity analysis to assess the association with dementia after exclusion of these subjects (*n*=15 807 including 14,667 subjects with cancer and 2738 immunocompromised subjects). We also further excluded the remaining subjects with at least one hospitalization related to herpesviruses (*n*=230), the severity of which could be associated with an increased risk of dementia and for which we do not know whether AHDs were given during hospitalization. Second, we also hypothesized that AHDs might be less prescribed for subjects with cognitive decline, in particular during the period just before the diagnosis of dementia. Thus, to avoid such reverse causality bias, we defined a 1-year lag-time (i.e., all treated subjects were considered as “unexposed” during the 1 year after AHD intake) [39], avoiding wrongly concluding that there is an increased risk of dementia in untreated subjects (in whom the absence of treatment might be precisely due to cognitive decline). Third, failing to specifically assess the impact of regular treatment on the risk of dementia, we rather excluded the few subjects with ≥ 2 deliveries of systemic AHDs per year of follow-up (*n*=169) to test their impact on the associations found.

All statistical tests were two-tailed, and the threshold for statistical significance was 5%. Analyses were performed using the statistical software SAS Enterprise Guide (version 9.4 SAS Institute, NC, USA).

## Results

### Characteristics of the included subjects

The characteristics of the included subjects are shown in Table 1. The mean age was 76 ± 8 years, and there were 41% men.

**Table 1:** Characteristics of the study sample according to the intake of systemic antiherpetic drugs. “Echantillon Généraliste des Bénéficiaires.” 2009–2017

	Study sample (***N***=68291)***N*** (%)	No systemic antiherpetics (***N***=61649)***N*** (%)	Systemic antiherpetics (***N***=6642) ***N*** (%)
Age at inclusion, mean ± standard deviation	76 ± 8	76 ± 8	74 ± 6
Sex - men	28286 (41.42)	25975 (42.13)	2311 (34.79)
Complementary health insurance for low-income people at inclusion	883 (1.29)	799 (1.30)	84 (1.26)
Comorbidities at inclusion			
Hypertension	40471 (59.26)	36400 (59.04)	4071 (61.29)
Diabetes	10584 (15.50)	9668 (15.68)	916 (13.79)
Heart disease	19753 (28.92)	17866 (28.98)	1887 (28.41)
Stroke	1294 (1.89)	1194 (1.94)	100 (1.51)
Hypercholesterolemia	25590 (37.47)	22737 (36.88)	2853 (42.95)
Intake of nonsteroidal anti-inflammatory drugs the year before inclusion			
0	43184 (63.24)	39695 (64.39)	3489 (52.53)
1 à 10	20246 (29.65)	17735 (28.77)	2511 (37.80)
≥10	4861 (7.12)	4219 (6.84)	642 (9.67)
Intake of systemic glucocorticoids the year before inclusion			
0	55301 (80.98)	50385 (81.73)	4916 (74.01)
1 à 10	12081 (17.69)	10465 (16.98)	1615 (24.31)
≥10	910 (1.33)	799 (1.30)	111 (1.67)
Intake of inhaled glucocorticoids the year before inclusion			
0	61175 (89.58)	55429 (89.91)	5746 (86.51)
1 à 10	5730 (8.39)	4975 (8.07)	755 (11.37)
≥10	1386 (2.03)	1245 (2.02)	141 (2.12)
Number of outpatient medical consultations the year before inclusion, median [IQR]	2 [0**–**6]	2 [0**–**6]	4 [1**–**8]
Number of different treatments the year before inclusion, median [IQR]	12 [6**–**18]	11 [5**–**18]	15 [9**–**21]

*Abbreviations*: *IQR* interquartile range

The proportion of subjects with at least one intake of AHDs during follow-up was 14.1% (*n*=9650), with 9.7% (*n*=6642) having systemic AHDs and 7.9% (*n*=5375) having nonsystemic AHDs (Table 2). Among subjects with at least one systemic AHD, 88.5% received at least one dose of valaciclovir and 16.6% at least one dose of aciclovir. Systemic AHDs were primary prescribed by general practitioners (80.4%), while ophthalmologists, dermatologists, hematologists/oncologists/internists, gynecologists, and neurologists prescribed only 9.1%, 3.7%, 3.3%, 0.9%, and 0.2% of treatments, respectively. Subjects with at least one intake of systemic AHDs were likely to be younger, more often women, more often consumers of lipid-lowering and anti-inflammatory drugs and to have a higher number of different treatments and medical consultations (Table 1).

**Table 2:** Intake of antiherpetic drugs during follow-up. ‶Echantillon Généraliste des Bénéficiaires.″ 2009–2017

	Study sample ***N***=68291, ***N*** (%)	Systemic antiherpetic drugs (***N***=6642), ***N*** (%)
**Antiherpetic drugs**	9650 (14.13)	
**Systemic antiherpetic drugs**	6642 (9.73)	6642 (100.00)
J05AB01 Aciclovir	1101 (1.61)	1101 (16.58)
J05AB09 Famciclovir	26 (0.04)	26 (0.39)
J05AB11 Valaciclovir	5879 (8.61)	5879 (88.51)
J05AB14 Valganciclovir	17 (0.02)	17 (0.26)
Number of deliveries during follow-up, median [IQR], p90 and p95		1 [1**–**2], 7 and 16
Number of deliveries per year of follow-up, median [IQR], p90 and p95		0.12 [0.11**–**0.33], 1 and 2.3
≥ 2 deliveries per year of follow-up	169 (0.25)	169 (2.54)
**Nonsystemic antiherpetic drugs**	5375 (7.87)	2367 (35.64)
**Dermatological application**	4903 (7.18)	2067 (31.12)
D06BB03 Aciclovir	4903 (7.18)	2067 (31.12)
**Ophthalmological application**	615 (0.90)	420 (6.32)
S01AD02 Trifluridine	93 (0.14)	39 (0.59)
S01AD03 Aciclovir	341 (0.50)	267 (4.02)
S01AD09 Ganciclovir	247 (0.36)	167 (2.51)

*Abbreviations*: *IQR* interquartile range, *p90* 90th percentile, *p95* 95th percentile

There were very few subjects with regular treatment (Table 2). Among those with at least one intake of systemic AHDs, the median number of deliveries during follow-up (mean time 7 ±3 years, median 9 years) was equal to 1, the third quartile to 2 and the 90th percentile to 7. It represents a median number of deliveries *per year of follow-up* of 0.12 (interquartile range (IQR) = 0.11–0.33) and, only 169 subjects (2.54% of the subjects with at least one AHD) had ≥ 2 deliveries of systemic AHDs per year of follow-up. Even among the few subjects with a particularly high number of deliveries during follow-up (≥7, *n*=665), the median number of deliveries per year of follow-up was limited (median 2.1, IQR = 1.2–4.1, min-max=0.8–24.1). This prevented us from further studying the association between regular treatment and the incidence of dementia.

Notably, the number of subjects with at least one hospitalization related to herpesviruses was also low: 0.22% of the subjects (*n*=151) had a hospitalization related to HSV infection (including 0.03% related to herpetic meningoencephalitis (*n*=23)), 0.02% (*n*=16) to CMV infection, and 0.36% (*n*=244) to VZV infection.

### Incident cases of dementia

During follow-up, 8883 subjects were identified as having incident dementia (5366 AD and 1784 VaD). Among AD cases, 974 (18.2%) were identified thanks to the presence of the three selected criteria (anti-dementia drugs, hospitalizations, and declaration of LTD related to AD), 1550 (28.9%) by two criteria, 925 (17.2%) only by the prescription of anti-dementia drugs, 525 (9.8%) only by a declaration of LTD related to AD, and 1392 (25.9%) only by an hospitalization related to AD. The incidence rates of dementia, AD and VaD were 18.2, 11.0, and 3.7 cases per 1000 person-years, respectively. The mean age at diagnosis of dementia was 84 ± 6 years. Among the 509 subjects treated with systemic AHDs and subsequently diagnosed with dementia, the median time interval between the first treatment and the occurrence of dementia was 2.9 years (IQR 1.2–4.8).

### Association between the intake of at least one antiherpetic drugs and onset of dementia

After adjustment for potential confounding factors, the intake of at least one systemic AHD was significantly associated with a decreased risk of AD (aHR 0.85 [0.75–0.96], *p*=0.009) and, to a lesser extent with respect to *p* values, with both dementia (aHR=0.90 [0.82–0.99], *p*=0.03) and VaD (aHR 0.80 [0.65–0.995], *p*=0.045) (Table 3).

**Table 3:** Association between intake of at least one systemic antiherpetic drugs and incidence of dementia - Cox models. ‶Echantillon Généraliste des Bénéficiaires.″ 2009–2017

	Adjusted model^a^			
	Events	aHR	95% CI	*P*-value
In all the subjects (*n*= 68291)				
All dementias	8883	**0.90**	**0.82–0.99**	**0.03**
Alzheimer’s disease	5366	**0.85**	**0.75–0.96**	**0.009**
Vascular dementia	1784	**0.80**	**0.65–0.995**	**0.045**
After exclusion of immunocompromised subjects, subjects with cancer or with an hospitalization related to herpesviruses (*n*=52254)				
All dementias	7044	**0.84**	**0.75–0.94**	**0.002**
Alzheimer’s disease	4260	**0.82**	**0.71–0.95**	**0.007**
Vascular dementia	1433	**0.77**	**0.60–0.99**	**0.04**
After carrying out a lag-time of 1 year (*n*= 68291)				
All dementias	8883	**0.86**	**0.78–0.95**	**0.004**
Alzheimer’s disease	5366	**0.78**	**0.68–0.90**	**0.001**
Vascular dementia	1784	0.87	0.69**–**1.09	0.23
After exclusion of participants with ≥ 2 deliveries of systemic AHDs per year of follow-up (*n*= 67904)				
All dementias	8856	**0.91**	**0.83–0.997**	**0.04**
Alzheimer’s disease	5351	**0.85**	**0.75–0.97**	**0.01**
Vascular dementia	1781	0.83	0.66**–**1.03	0.09

*Abbreviations*: *aHR* adjusted hazard ratios, *95% CI* 95% confidence interval, *AHD* antiherpetic drug

^**a**^Adjustment for age at inclusion, sex, being beneficiary of a complementary health insurance for low-income people at inclusion, hypertension, diabetes, hypercholesterolemia, heart disease, stroke, intake of nonsteroidal anti-inflammatory drugs, systemic, or inhaled glucocorticoids the year before inclusion, number of different medications the year before inclusion, number of outpatient medical consultations the year before inclusion

Results of the sensitivity analyses are shown in Table 3. First, the exclusion of subjects with cancer, immunocompromised subjects or subjects with at least one hospitalization related to herpesviruses did not profoundly change the results. Second, after the introduction of a lag time, the associations remained significant for dementia from any cause and AD but not for VaD. Third, the observed association did not seem to be solely based on a potential protective effect of a “regular” treatment in the few subjects with ≥ 2 deliveries of systemic AHDs per year of follow-up because, after their exclusion, the results were similar to those of the main analysis.

## Discussion

### Main results

First, intake of at least one systemic AHD was significantly associated with a 15% reduction in the risk of developing AD and, to a lesser extent with respect to *p* values, to both all-cause dementia and VaD. This association remained after exploring various potential biases. Second, there were very few subjects with regular treatment, preventing the assessment of a potential association between regular treatment and the incidence of dementia.

### Interpretation in light of literature

#### Previous results

Different studies have previously explored the question of an effect of AHDs on the risk of dementia with various methodologies: a comparison of methods used and the results obtained are available in Supplemental Table 1.

Using a Taiwanese medico-administrative database, Tzeng et al. [28] first studied the question in a very particular population: subjects over 50 and defined as “with newly diagnosed HSV infection” if they had at least three outpatient visits related to HSV-1 or HSV-2 infections in the inclusion year and no visit related to HSV previously. Among them, subjects treated with AHDs had a very marked decrease in the risk of dementia (aHR 0.092 [0.079–0.108], *p*< 0.001), suggesting that 90% of dementia cases in this population could be prevented by AHDs. Notably, the association remained significant regardless of the duration of treatment considered (< or ≥ 30 days of treatment during the follow-up). The discrepancy between Tzeng’s results and ours regarding the magnitude of the association may be partially due to the selection of subjects: Tzeng et al. included subjects with potentially more severe infections as reflected by the high prevalence of subjects treated (86%) and the use in some cases of intravenous treatments (ex: gancicolovir). In addition, the majority of incident dementias in this study were neither Alzheimer’s diseases nor vascular dementias. It questions the proportion of purely infectious dementias in this study (linked to Herpes viruses or to infections favoring their reactivation such as HIV) which could explain a significant part of the protective association found.

Subsequently, other studies found results of a magnitude more consistent with ours. In two medico-administrative databases from Taiwan [29] and South Korea [30], subjects diagnosed with Herpes Zoster (due to VZV infection) and treated at least once by AHDs had a lower risk of dementia compared to diagnosed and untreated subjects (aHR=0.55 [0.40–0.77], *p* < 0.001 and aHR=0.76 [0.65–0.90], *p*=0.001, respectively). In Sweden [31], subjects diagnosed with HSV or VZV and treated at least once by AHDs had a lower risk of dementia compared to undiagnosed and untreated subjects (aHR=0.90 [0.82–0.98], *p*=0.015). Similar associations persisted when comparing (i) treated subjects (regardless of whether or not a diagnosis of infection existed) to undiagnosed and untreated subjects (aHR=0.89 [0.86–0.92], *p*<0.001) and (ii) diagnosed and treated subjects to diagnosed and untreated subjects (aHR=0.75 [0.68–0.83], *p*<0.001). Also from Sweden, a nested case-control study [32] was carried out in the BETULA cohort in order to benefit from serological and genetic data. Thus, among subjects infected with HSV-1 (i.e., anti-HSV1 IgG-positive subjects), AD cases had less frequently an history of AHD prescription compared to controls matched by age, sex, study sample start year, and APOEε4 (OR=0.287 [0.102–0.809], *p*=0.018). Finally, Schnier et al. [33] studied the question in four European databases and found discordant results. Thus, in the Danish database, treated subjects had a lower risk of dementia compared to untreated subjects (aHR=0.91 [0.89–0.93], *p*<0.001 for subjects treated once, aHR=0.93 [0.88–0.98], *p*=0.008 for subjects treated twice, aHR=0.89 [0.83–0.95] *p*<0.001 for subjects treated three or more times during follow-up). In our opinion, the absence of a dose effect is not so surprising given that the intake of two or even three AHDs is far from representing a regular treatment over a period of several years. In the Welsh database, a similar association was found only for the subjects diagnosed with Herpes simplex or Herpes zoster and treated once compared to undiagnosed and untreated subjects (aHR=0.91 [0.86–0.97], *p*=0.002) while associations were no longer significant when (i) dementia subtypes were studied separately (aHR=0.91 [0.84–1.00] for AD and aHR=0.95 [0.86–1.05] for VaD), potentially linked to difficulties in identifying dementia subtypes in medico-administrative databases and (ii) for subjects treated twice or more, potentially reflecting the smaller numbers in these categories. At last, in the Scottish and German databases, no association was found but, in these databases (as well as in the Welsh database), analyses were only adjusted on age, sex, and some proxy of social deprivation leaving a risk of residual confounding bias.

#### Interpretation of our results

We highlighted a reduced risk of dementia in subjects taking at least one systemic AHD during the follow-up period. Nevertheless, our results should be interpreted with caution given the low frequency of subjects receiving regular treatment. Indeed, although it seems implausible that a single intake of systemic AHDs has a protective effect on AD, the association found (here and in most of the previous studies) could nevertheless reflect the impact of a more regular intake of AHDs prior to the inclusion date.

An alternative hypothesis would be that the occurrence of peripheral reactivations (and their possible subsequent treatment) is negatively correlated with the occurrence of central reactivations secondary to the migration of the virus into the central nervous system (and therefore to the risk of dementia). This negative correlation between peripheral and central reactivations could be supported by the fact that some studies based on self-reported cold sores described a counterintuitive reduction in HSV reactivations with age [40, 41], while other studies based on serological parameters suggest the opposite occurs [42, 43].

Finally, the association could be due to some residual methodological biases. However, in our study, unparalleled efforts have been made to limit their impact. Regarding confounding bias, we controlled for numerous factors including (i) sociodemographic data; (ii) comorbidities which are known risk factors of AD (and whose link with taking AHD is unknown), and not those which could be first signs of dementia; (iii) intake of anti-inflammatory drugs (that might impact both viral reactivation and the development of dementia); (iv) the number of different treatments taken (which could have an impact on the decision to prescribe AHDs); and (v) proxy of health care use (i.e., number of medical consultations in the year before inclusion, which impacts both the likelihood of being treated and being diagnosed). Note that we were unable to take into account some genetic risk factors. However, as the main genetic risk of AD, APOE4, is considered a risk factor for both cold sores and herpes zoster [44, 45] and is therefore potentially associated with more frequent use of AHDs, its absence would have underestimated the protective association observed rather than overestimated the association. To avoid an indication bias, we also excluded the participants for whom the probability of being identified as demented could be modified by the pathology that led to the prescription of AHDs. We also performed a 1-year lag-time to avoid a reverse causality bias. Note that the relatively short delay between the intake of systemic AHD and onset of dementia in our sample (median 2.9 years) prevented us from achieving a longer lag-time (which would have been better considering the long prodromal phase of AD [46, 47]).

### Limitations

The main strengths and limits of our study are inherent in the use of medico-administrative databases. Such a data source allows analyses on a large study sample and therefore high statistical power. It is also representative of the national population in terms of sex and age and benefits from a relatively long follow-up with no loss to follow-up and from exhaustive data on hospitalizations, out-hospital drug reimbursements and death recordings.

One of the limitations of our study is the inability to distinguish between individuals infected with HSV and those uninfected. Indeed, AHDs are only a good marker of the treated infection and not of HSV infection in general. While a large proportion of elderly subjects are infected with HSV-1 (approximately 80% (10)), only approximately 20% exhibit symptomatic reactivations [48], and an even smaller proportion are treated with AHDs. Consequently, due to the lack of additional clinical and paraclinical information, the unexposed group includes both infected subjects and, to a lesser extent, uninfected subjects, which could have underestimated the association identified. In addition, the lack of information on the indication of AHDs has made it impossible to clearly distinguish between orofacial herpes (primarily due to HSV-1), genital herpes (primarily due to HSV-2), or other symptoms related to CMV or VZV infections. However, whatever the indication for treatment, systemic AHDs will have been effective on the majority of herpes viruses infecting the subject and in particular on HSV, which has a high prevalence. Finally, due to a too low proportion of subjects with regular intake of AHDs, the impact of regular treatment could not be evaluated.

With respect to the detection of dementia, while the number of dementia cases is underestimated due to the underdiagnosis of dementia in the general population and the lack of specific care for some of the diagnosed individuals (i.e., anti-dementia drugs or LTD), the identification of demented subjects in the EGB was improved by the use of primary and secondary diagnoses related to any hospitalizations in medical and surgical wards. Moreover, an insufficient detection of demented subjects due to non-recourse to care for financial reasons seems limited knowing that (i) almost the entire French population has a health insurance and (ii) the majority of medical costs related to the onset of dementia is, to varying degrees, subject to reimbursement. Therefore, the incidence of dementia in our study is not very different from that reported in the literature (18.39 cases per 1000 person-years in high-income countries for persons over 60 [49]). Finally, analyses by dementia subtype should be interpreted with caution given that (i) the quality of the ICD10 codes associated with hospitalizations and LTD is probably not sufficient to allow accurate determination of the etiology of dementia; (ii) in some clinical settings, it is likely that only clinical data have been available to determine the etiology of dementia; and (iii) “mixed” dementias are frequent. Nevertheless, in this study, efforts have been made to improve the validity of the diagnosis using a combination of three identification criteria (i.e., treatment, LTD, and hospitalization). Thus, in our sample, 47.1% of AD cases were identified using two or three criteria and another 17.2% were identified using anti-dementia drugs (mainly prescribed for AD).

The strengths and limitations of statistical analyses have been discussed in paragraph 4.2.2.

## Conclusions

First, taking at least one systemic AHD during follow-up was significantly associated with a 15% reduced risk of developing AD, even after taking into account several potential methodological biases. Nevertheless, our results should be interpreted with caution given the low frequency of subjects receiving *regular* treatment and could reflect either a protective effect of systemic AHDs on the development of dementia or residual biases. Second, our results highlight the limits of epidemiological databases for studying the association between a *regular* treatment and the incidence of dementia. Thus, given the growing body of evidence supporting the implication of herpesviruses in the onset of dementia and the dire lack of effective treatments for the prevention of AD, data of clinical trials with prolonged treatment with systemic AHDs are awaited to evaluate these easily accessible, well-tolerated and inexpensive treatments as preventive measures against dementia. Two phase 2 clinical trials are currently ongoing. One Swedish open pilot clinical trial (NCT02997982) is testing the effect of 4 weeks of oral valaciclovir in 36 APOE4 carriers with AD or with amnesic mild cognitive impairment. One American double-blind randomized trial (NCT03282916) is comparing the effect of valaciclovir for 18 months in 65 treated participants and 65 controls, all with mild AD. Results from these first trials will probably need to be extended to an earlier stage of the disease in larger preventive clinical trials.

## Supplementary Information

**Additional file 1: **Definitions of exposition, incidence of dementia and other variables*.* **Additional table 1**: Comparison of previous human studies assessing the impact of antiherpetic drugs on the onset of dementia*.*

## Data Availability

Publicly sharing EGB data is forbidden by law according to The French National Data Protection Agency (Commission Nationale de l’Informatique et des LIbertés, CNIL); regulatory decisions AT/CPZ/SVT/JB/DP/CR05222O of June 14, 2005, and DP/CR071761 of August 28, 2007. To request data access please contact The National Institute for Health Data (Institut National des Données de Santé, INDS).

## Abbreviations

AD: Alzheimer’s disease
AHD: Antiherpetic drug
LTD: Long-term diseases (a group of diseases for which all medical expenses are fully reimbursed)
ATC: Anatomical Therapeutic Chemical
CI: Confidence interval
CMV: Cytomegalovirus
EGB: “*Echantillon Généraliste des Bénéficiaires*”
aHR: Adjusted hazard ratio
HSV-1: Herpes simplex virus type 1
HSV-2: Herpes simplex virus type 2
ICD: International Classification of Diseases
VaD: Vascular dementia
VZV: Varicella zoster virus

## References

[1] Soscia SJ, Kirby JE, Washicosky KJ, Tucker SM, Ingelsson M, Hyman B (2010). The Alzheimer’s disease-associated amyloid β-protein is an antimicrobial peptide. PLoS One.

[2] Moir RD, Lathe R, Tanzi RE (2018). The antimicrobial protection hypothesis of Alzheimer’s disease. Alzheimers Dement.

[3] Bourgade K, Dupuis G, Frost EH, Fülöp T (2016). Anti-viral properties of amyloid-β peptides. J Alzheimers Dis.

[4] Gosztyla ML, Brothers HM, Robinson SR. Alzheimer’s amyloid-b is an antimicrobial peptide: a review of the evidence. J Alzheimers Dis. 2018;62(4):1495-506. 10.3233/JAD-171133. 10.3233/JAD-171133 29504537

[5] Readhead B, Haure-Mirande J-V, Funk CC, Richards MA, Shannon P, Haroutunian V (2018). Multiscale analysis of independent Alzheimer’s cohorts finds disruption of molecular, genetic, and clinical networks by human herpesvirus. Neuron..

[6] Tsai M-C, Cheng W-L, Sheu J-J, Huang C-C, Shia B-C, Kao L-T (2017). Increased risk of dementia following herpes zoster ophthalmicus. PLoS One.

[7] Barnes LL, Capuano AW, Aiello AE, Turner AD, Yolken RH, Torrey EF (2015). Cytomegalovirus infection and risk of alzheimer disease in older black and white individuals. J Infect Dis.

[8] Itzhaki RF (2018). Corroboration of a major role for herpes simplex virus type 1 in Alzheimer’s disease. Front Aging Neurosci.

[9] Steel AJ, Eslick GD (2015). Herpes viruses increase the risk of Alzheimer’s disease: a meta-analysis. J Alzheimers Dis.

[10] Looker K, Garnett G (2005). A systematic review of the epidemiology and interaction of herpes simplex virus types 1 and 2. Sex Transm Infect.

[11] Miller CS, Danaher RJ (2008). Asymptomatic shedding of herpes simplex virus (HSV) in the oral cavity. Oral Surg Oral Med Oral Pathol Oral Radiol Endod.

[12] Harris SA, Harris EA. Molecular mechanisms for herpes simplex virus type 1 pathogenesis in Alzheimer’s disease. Front Aging Neurosci. 2018;10:48. 10.3389/fnagi.2018.00048. eCollection 2018. 10.3389/fnagi.2018.00048 PMC5845560 29559905

[13] De Chiara G, Piacentini R, Fabiani M, Mastrodonato A, Marcocci ME, Limongi D (2019). Recurrent herpes simplex virus-1 infection induces hallmarks of neurodegeneration and cognitive deficits in mice. PLoS Pathog.

[14] Streit WJ, Xue Q-S (2014). Human CNS immune senescence and neurodegeneration. Curr Opin Immunol.

[15] McManus RM, Heneka MT (2017). Role of neuroinflammation in neurodegeneration: new insights. Alz Res Therapy.

[16] Carter C. Alzheimer’s disease: APP, gamma secretase, APOE, CLU, CR1, PICALM, ABCA7, BIN1, CD2AP, CD33, EPHA1, and MS4A2, and their relationships with herpes simplex, C. pneumoniae, other suspect pathogens, and the immune system. Int J Alzheimers Dis. 2011. 10.4061/2011/501862 PMC3255168 22254144

[17] Carter CJ (2011). Alzheimer’s disease plaques and tangles: cemeteries of a pyrrhic victory of the immune defence network against herpes simplex infection at the expense of complement and inflammation-mediated neuronal destruction. Neurochem Int.

[18] Carter CJ (2008). Interactions between the products of the herpes simplex genome and Alzheimer’s disease susceptibility genes: relevance to pathological-signalling cascades. Neurochem Int.

[19] Itzhaki RF, Lin W-R (1998). Herpes simplex virus type I in brain and the type 4 allele of the apolipoprotein E gene are a combined risk factor for Alzheimer’s disease. Biochem Soc Trans.

[20] Itzhaki R, Wozniak M (2006). Herpes simplex virus type 1, apolipoprotein E, and cholesterol: a dangerous liaison in Alzheimer’s disease and other disorders. Prog Lipid Res.

[21] Urosevic N, Martins RN (2008). Infection and Alzheimer’s disease: the APOE ε4 connection and lipid metabolism. J Alzheimers Dis.

[22] Linard M, Letenneur L, Garrigue I, Doize A, Dartigues J-F, Helmer C (2020). Interaction between APOE4 and herpes simplex virus type 1 in Alzheimer’s disease. Alzheimers Dement.

[23] Linard M, Baillet M, Letenneur L, Garrigue I, Catheline G, Dartigues J-F (2021). Herpes simplex virus, early neuroimaging markers and incidence of Alzheimer’s disease. Transl Psychiatry.

[24] Wozniak MA, Frost AL, Preston CM, Itzhaki RF (2011). Antivirals reduce the formation of key Alzheimer’s disease molecules in cell cultures acutely infected with herpes simplex virus type 1. PLoS One.

[25] Lukiw WJ, Cui JG, Yuan LY, Bhattacharjee PS, Corkern M, Clement C (2010). Acyclovir or Aβ42 peptides attenuate HSV-1-induced miRNA-146a levels in human primary brain cells. NeuroReport..

[26] Zambrano Á, Solis L, Salvadores N, Cortés M, Lerchundi R, Otth C (2008). Neuronal cytoskeletal dynamic modification and neurodegeneration induced by infection with herpes simplex virus type 1. J Alzheimers Dis.

[27] Powell-Doherty RD, Abbott ARN, Nelson LA, Bertke AS (2020). Amyloid-β and p-tau anti-threat response to herpes simplex virus 1 infection in primary adult murine hippocampal neurons. J Virol.

[28] Tzeng N-S, Chung C-H, Lin F-H, Chiang C-P, Yeh C-B, Huang S-Y (2018). Anti-herpetic medications and reduced risk of dementia in patients with herpes simplex virus infections—a nationwide, population-based cohort study in Taiwan. Neurotherapeutics..

[29] Chen VC-H, Wu S-I, Huang K-Y, Yang Y-H, Kuo T-Y, Liang H-Y, et al. Herpes zoster and dementia: a nationwide population-based cohort study. J Clin Psychiatry. 2018;79(1). 10.4088/JCP.16m11312 29244265

[30] Bae S, Yun S-C, Kim M-C, Yoon W, Lim JS, Lee S-O, et al. Association of herpes zoster with dementia and effect of antiviral therapy on dementia: a population-based cohort study. Eur Arch Psychiatry Clin Neurosci. 2020. 10.1007/s00406-020-01157-4 32613564

[31] Lindman KL, Hemmingsson E-S, Brännström J, Josefsson M, Olsson J, Nordström P (2021). Herpesvirus infections, antiviral treatment, and the risk of dementia—a registry-based cohort study in Sweden. Alzheimer’s Dement Transl Res Clin Intervent.

[32] Hemmingsson E, Hjelmare E, Weidung B, Olsson J, Josefsson M, Adolfsson R, et al. Antiviral treatment associated with reduced risk of clinical Alzheimer’s disease—a nested case-control study. Alzheimer’s Dement Transl Res Clin Intervent. 2021. 10.1002/trc2.12187 PMC8190532 34136638

[33] Schnier C, Janbek J, Williams L, Wilkinson T, Laursen TM, Waldemar G (2021). Antiherpetic medication and incident dementia: observational cohort studies in four countries. Eur J Neurol.

[34] Bezin J, Duong M, Lassalle R, Droz C, Pariente A, Blin P (2017). The national healthcare system claims databases in France, SNIIRAM and EGB: powerful tools for pharmacoepidemiology. Pharmacoepidemiol Drug Saf.

[35] Tuppin P, de Roquefeuil L, Weill A, Ricordeau P, Merlière Y (2010). French national health insurance information system and the permanent beneficiaries sample. Rev Epidemiol Sante Publique.

[36] Palmaro A, Moulis G, Despas F, Dupouy J, Lapeyre-Mestre M (2016). Overview of drug data within French health insurance databases and implications for pharmacoepidemiological studies. Fundam Clin Pharmacol.

[37] Schneeweiss S, Rassen JA, Brown JS, Rothman KJ, Happe L, Arlett P (2019). Graphical depiction of longitudinal study designs in health care databases. Ann Intern Med.

[38] Kaufman HE, Azcuy AM, Varnell ED, Sloop GD, Thompson HW, Hill JM (2005). HSV-1 DNA in tears and saliva of normal adults. Invest Opthalmol Visual Sci.

[39] Pottegård A, Friis S, Stürmer T, Hallas J, Bahmanyar S (2018). Considerations for pharmacoepidemiological studies of drug-cancer associations. Basic Clin Pharmacol Toxicol.

[40] Lorette G, Crochard A, Mimaud V, Wolkenstein P, Stalder J-F, El Hasnaoui A (2006). A survey on the prevalence of orofacial herpes in France: the INSTANT study. J Am Acad Dermatol.

[41] Malvy D, Ezzedine K, Lançon F, Halioua B, Rezvani A, Bertrais S (2007). Epidemiology of orofacial herpes simplex virus infections in the general population in France: results of the HERPIMAX study. J Eur Acad Dermatol Venereol.

[42] Stowe RP, Peek MK, Cutchin MP, Goodwin JS (2012). Reactivation of herpes simplex virus type 1 is associated with cytomegalovirus and age. J Med Virol.

[43] Stowe R, Kozlova E, Yetman D, Walling D, Goodwin J, Glaser R (2007). Chronic herpesvirus reactivation occurs in aging. Exp Gerontol.

[44] Kunkle BW, Grenier-Boley B, Alzheimer Disease Genetics Consortium (ADGC), The European Alzheimer’s Disease Initiative (EADI), Cohorts for Heart and Aging Research in Genomic Epidemiology Consortium (CHARGE), Genetic and Environmental Risk in AD/Defining Genetic, Polygenic and Environmental Risk for Alzheimer’s Disease Consortium (GERAD/PERADES) (2019). Genetic meta-analysis of diagnosed Alzheimer’s disease identifies new risk loci and implicates Aβ, tau, immunity and lipid processing. Nat Genet.

[45] Itzhaki RF, Wozniak MA. Herpes simplex virus type 1 in Alzheimer’s disease: the enemy within. J Alzheimers Dis. 2008;13. 10.3233/jad-2008-13405 18487848

[46] Amieva H, Mokri H, Le Goff M, Meillon C, Jacqmin-Gadda H, Foubert-Samier A (2014). Compensatory mechanisms in higher-educated subjects with Alzheimer’s disease: a study of 20 years of cognitive decline. Brain..

[47] Bezin J, Bosco-Levy P, Pariente A (2017). False-positive results in pharmacoepidemiology and pharmacovigilance. Therapies..

[48] Forbes H, Warne B, Doelken L, Brenner N, Waterboer T, Luben R (2019). Risk factors for herpes simplex virus type-1 infection and reactivation: cross-sectional studies among EPIC-Norfolk participants. PLoS One.

[49] World Alzheimer Report (2015). The global impact of dementia: an analysis of prevalence, incidence, cost and trends.

