# Supplementary material for: Antiherpetic drugs: a potential way to prevent Alzheimer’s disease?
Source: Alzheimers Res Ther. 2022 Jan 7;14:3. doi: 10.1186/s13195-021-00950-0 (PMC8742322; doi:10.1186/s13195-021-00950-0)
Supplement: Supplementary file 1 — Additional file 1: Definitions of exposition, incidence of dementia and other variables. Additional table 1: Comparison of previous human studies assessing the impact of antiherpetic drugs on the onset of dementia. [file 13195_2021_950_MOESM1_ESM.zip › Additional file.docx]

## **Additional file**

## Additional table 1: Comparison of previous human studies assessing the impact of antiherpetic drugs on the onset of dementia

See Excel file.

## Additional file 1: Definitions of exposition, incidence of dementia and other variables

**Exposition**

The ATC codes used to identify the intake of antiherpetic drugs are as follows:

| **Antiherpetics with systemic action** | **Antiherpetics for ophthalmological use** | **Antiherpetics for dermatological use** |
| --- | --- | --- |
| J05AB01 Aciclovir  J05AB02 Idoxuridine  J05AB03 Vidarabine  J05AB06 Ganciclovir  J05AB09 Famciclovir  J05AB11 Valaciclovir  J05AB12 Cidofovir  J05AB13 Penciclovir  J05AB14 Valganciclovir  J05AB15 Brivudine  J05AC03 Tromantadine  J05AD01 Foscarnet  J05AD02 Fosfonet | S01AD01 Idoxuridine  S01AD02 Trifluridine  S01AD03 Aciclovir  S01AD07 Famciclovir  S01AD09 Ganciclovir | D06BB02 Tromantadine  D06BB03 Aciclovir  D06BB08 Ibacitabine  D06BB11 Docosanol |

The ICD-10 codes used to identify the occurrence of a hospitalization related to HSV, CMV or VZV infections are as follows:

- Hospitalization related to HSV
  - (B00) Infections with the herpes virus (herpes simplex)
    - (B00.0) Herpetic eczema
    - (B00.1) Vesicular dermatitis due to the herpes virus
    - (B00.2) Gingivostomatitis and pharyngotonsillitis due to the herpes virus
    - (B00.3) Meningitis due to the herpes virus
    - (B00.4) Encephalitis due to the herpes virus
    - (B00.5) Eye disorders caused by the herpes virus
    - (B00.7) Disseminated disease caused by the herpes virus
    - (B00.8) Other forms of infection due to the herpes virus
    - (B00.9) Infection due to the herpes virus, unspecified
  - (A60) Anogenital infection with the herpes virus (herpes simplex)
    - (A60.0) Infection of the genitals and genitourinary system with the herpes virus
    - (A60.1) Infection of the cutaneous margin of the anus and rectum, by the herpes virus
    - (A60.9) Anogenital infection with the herpes virus, unspecified
- Hospitalization in connection with other herpesviruses
  - VZV
    - (B01) Chickenpox
    - (B02) Shingles
  - CMV
    - (B20.2) HIV disease causing cytomegalovirus infections
    - (B25) Cytomegalovirus disease
      - (B25.0) Cytomegalovirus pneumonia
      - (B25.1) Cytomegalovirus hepatitis
      - (B25.2) Cytomegalovirus pancreatitis
      - (B25.8) Other cytomegalovirus diseases
      - (B25.9) Cytomegalovirus disease, unspecified

**Incidence of dementia**

The following criteria were used for the identification of prevalent and incident dementias:

|  | Dementia from any cause | Alzheimer’s disease | Vascular dementia |
| --- | --- | --- | --- |
| Hospitalizations with ICD10 codes | F0, G30, F01, F02, F03, G31, G32 | F0 G30 | F01 |
| Long-term disease related to dementia | LTD n° 15 “Alzheimer's disease and other dementias”  + ICD10 codes: F0, G30, F01, F02, F03, G31, G32 | LTD n° 15  + ICD10 codes F0, G30 | LTD n° 15  + ICD10 codes F01 |
| ATC codes for anti-dementia drugs | Anti-cholinesterase drugs N06DA02 N06DA03 N06DA04  Memantine N06DX01  Their association N06DA52 N06DA53 | | None |

With ICD10 codes corresponding to:

- (F00.0) Dementia in Alzheimer's disease, early onset

- (F00.1) Dementia in Alzheimer's disease, late onset

- (F00.2) Dementia in Alzheimer's disease, atypical or mixed form

- (F00.9) Dementia in Alzheimer's disease, unspecified

- (G30 [archive]) Alzheimer's disease

- (F01.0) Acute onset vascular dementia

- (F01.1) Vascular dementia due to multiple infarctions

- (F01.2) Subcortical vascular dementia

- (F01.3) Mixed vascular dementia, cortical and subcortical

- (F01.8) Other forms of vascular dementia

- (F01.9) Vascular dementia, unspecified

- (F02.0) Dementia in Pick's disease

- (F02.1) Dementia in Creutzfeldt-Jakob disease

- (F02.2) Dementia in Huntington's disease

- (F02.3) Dementia in Parkinson's disease

- (F02.4) Dementia due to human immunodeficiency virus (HIV) disease

- (F02.8) Dementia in other diseases classified elsewhere

- (F03) Dementia, unspecified

- (G31 [archive]) Other degenerative disorders of the nervous system, not elsewhere classified

- (G32 [archive]) Other degenerative disorders of the nervous system in disorders classified elsewhere

**Adjustment variables**

The presence of comorbidities was defined considering either the declaration of a long-term disease (LTD) before inclusion or the intake of medications the year before inclusion as following:

|  | **Declaration of a long-term disease** | **Intake of medication with the following ATC codes** |
| --- | --- | --- |
| **Hypertension** | LTD N°12 “Severe hypertension” | Anti-hypertensives C02 C03 C07 C08 C09 |
| **Diabetes** | LTD N°8 “Type 1 and type 2 diabetes” | Anti-diabetics A10 |
| **Stroke** | LTD N°1 “Disabling stroke” |  |
| **Heart disease** | LTD N°3 “Chronic arteriopathies with ischemic manifestations”  ± LTD N°13 “Coronary disease”  ± LTD N°5 “Severe heart failure, severe arrhythmia, severe valvular heart disease, severe congenital heart disease” | Antiplatelet drugs B01AC |
| **Hypercholesterolemia** |  | Lipid-lowering C10AA C10B C10AB |

The ATC codes considered for the identification of taking anti-inflammatory drugs the year before inclusion were the following: M01A MO1B for nonsteroidal anti-inflammatory drugs, H02AB H02B M01BA for systemic glucocorticoids, R03BA R03AL09 R03AK06 R03AK07 R03AK08 R03AK09 R03AK10 R03AK11 R03AK12 R03AK13 for inhaled glucocorticoids. Then, categorical variables were created to differentiate i) no intake, ii) non-regular intake or iii) regular intake defined as ≥10 deliveries in the year preceding inclusion.

The number of different medications (defined by their ATC codes) before inclusion and the number of medical consultations before inclusion were defined as categorical variables using terciles (0, 1-3, 3-6, ≥6 consultations and 0, 1-9, 9-15 and ≥ 15 different medications).

**Variables used in sensitivity analyses**

The following variables (identified before inclusion or during follow-up) were used to perform sensitivity analyses:

|  | **Declaration of a long-term disease** | **Intake of medication with the following ATC codes** |
| --- | --- | --- |
| Immunocompromised subjects | - LTD N°2 “Bone marrow failure and other chronic cytopenias” with ICD10 codes associated with medullar aplasia or myelodysplastic syndromes  - LTD N° 7 “Severe primary immune deficiency requiring prolonged treatment, infection with human immunodeficiency virus”  - LTD N°28 “Organ transplant suite” | - HIV treatments: J05AR J05AE J05AF J05AG  - Immunosuppressants or immunomodulators: L03 L04 |
| Subjects with cancer | - LTD N° 30 “Cancer” |  |
